# Fast Fourier single-pixel imaging via binary illumination

**DOI:** 10.1038/s41598-017-12228-3

**Published:** 2017-09-20

**Authors:** Zibang Zhang, Xueying Wang, Guoan Zheng, Jingang Zhong

**Affiliations:** 1 Department of Optoelectronic Engineering, Jinan University, Guangzhou, 510632 China; 2 Biomedical Engineering, University of Connecticut, Storrs, CT 06269 USA; 3 Guangdong Provincial Key Laboratory of Optical Fiber Sensing and Communications, Jinan University, Guangzhou, 510632 China

## Abstract

Fourier single-pixel imaging (FSI) employs Fourier basis patterns for encoding spatial information and is capable of reconstructing high-quality two-dimensional and three-dimensional images. Fourier-domain sparsity in natural scenes allows FSI to recover sharp images from undersampled data. The original FSI demonstration, however, requires grayscale Fourier basis patterns for illumination. This requirement imposes a limitation on the imaging speed as digital micro-mirror devices (DMDs) generate grayscale patterns at a low refreshing rate. In this paper, we report a new strategy to increase the speed of FSI by two orders of magnitude. In this strategy, we binarize the Fourier basis patterns based on upsampling and error diffusion dithering. We demonstrate a 20,000 Hz projection rate using a DMD and capture 256-by-256-pixel dynamic scenes at a speed of 10 frames per second. The reported technique substantially accelerates image acquisition speed of FSI. It may find broad imaging applications at wavebands that are not accessible using conventional two-dimensional image sensors.

## Introduction

Single-pixel imaging^1–31^ is a computational imaging scheme that allows an image to be captured using a detector without spatial resolution. Different from conventional single-shot imaging scheme, single-pixel imaging reconstructs the image computationally with multiple measurements. Early single-pixel imaging techniques perform raster scanning in the spatial domain to acquire the spatial information of objects. Laser confocal scanning microscopy^1^, optical coherence tomography^2^, ultrasonic endoscope^3^, and scanning electron microscopy^4^ are examples of single-pixel imaging which perform raster scanning in the spatial domain. Contemporary single-pixel imaging implementation employs spatial light modulators (SLMs) to generate intensity patterns for illumination. It shares its roots with ghost imaging^5–10^, which was initially considered as a quantum effect^5^ and later was implemented using a classical source^6^. Computational ghost imaging^7–10^ used an SLM to generate structured illumination and a single-pixel detector to capture the back-scattered light intensity which carries the spatial information of the scene. There are two types of single-pixel imaging configuration. The first one is passive single-pixel imaging which uses an SLM to control detection light fields. The other one is active single-pixel imaging which uses an SLM to control illumination light fields. Interestingly, active single-pixel imaging techniques are subject to Helmholtz reciprocity^32^. Consequently, the field of view is determined by the illumination unit while the shading profile is set by the detection unit. The scheme of single-pixel imaging allows one to build an imaging system with high signal-to-noise ratio, wide spectral range, and low cost in terms of detection units. Therefore, single-pixel imaging has attracted considerable attentions and been applied in terahertz imaging^11,12^, three-dimensional (3-D) imaging^13–18^, multispectral or hyperspectral imaging^19,20^, microcopy^21–23^, optical encryption^24^, imaging through turbid media^25^, remote sensing^26,27^, etc.

Mathematically, active single-pixel imaging reconstructs the image of an object by obtaining the inner products of the pixelated reflectivity distribution of the object with the illumination patterns. As single-pixel detectors give a single value in each measurement, single-pixel systems typically need to take a number of measurements for obtaining sufficient spatial information for reconstructing a sharp image. Single-pixel imaging, therefore, is essentially a time-multiplexed technique and the temporal resolution imposes a limit for many imaging applications. The acquisition time *t*_*A*_ depends on both the number of measurements *M* and the measurement rate *R*: *t*_*A*_ = *M*/*R*. To reduce the acquisition time, one can increase the measurement rate and reduce the number of measurements. The measurement rate *R* of a single-pixel imaging system is mainly determined by the illumination patterns generation rate. The latest digital micro-mirror devices (DMDs) are able to generate ~20,000 binary patterns per second. DMDs are much faster than conventional SLMs such as liquid crystal displays. Multiplexing techniques^28^ promise further acceleration but are at the expense of additional devices. Therefore, the straightforward solution is to maximize the pattern illumination rate given by the utilized DMD. The number of measurements needed mainly depends on the number of effective reconstructed pixels. The utilization of generic compressive sensing (CS) algorithms^11,12,14,29^ or the prior knowledge of object images allows reduction of the number of measurements. However, CS algorithms are generally computationally exhausted and cannot be implemented in real time.

For single-pixel imaging, both high image reconstruction quality and short image acquisition time are desirable. Recently, a single-pixel imaging technique, termed Fourier single-pixel imaging (FSI), was reported for producing high-quality two-dimensional (2-D)^30^ and 3-D images^17^. This technique uses grayscale Fourier basis patterns for illumination and acquires the Fourier spectrum of the object image. The speed of this technique is limited by the grayscale-pattern-generating rate of the employed DMD. Since DMDs generate each grayscale pattern by switching multiple binary patterns sequentially, the original FSI technique is inherently slow and it is challenging to apply it in high-speed imaging systems. On the contrary, Hadamard single-pixel imaging^15,19,29,31^ is well compatible with DMDs because of the binarized nature of the Hadamard basis patterns. Thus, it is relatively easy to achieve real-time single-pixel imaging using Hadamard patterns and impressive results have been reported in the past years. Recently, we compare the performances of Hadamard single-pixel imaging and Fourier single-pixel imaging with theoretical analysis and experiments^32^. The results show that Fourier single-pixel is more efficient than Hadamard single-pixel imaging in terms of the number of measurements. Therefore, it is also essential to adapt the original Fourier single-pixel imaging to a DMD-based imaging system so that high-quality and fast single-pixel imaging can be achieved.

In this paper, we convert the Fourier basis patterns from grayscale to binary by using the Floyd-Steinberg error diffusion dithering method^33^. The binarized Fourier basis patterns allow us to utilize high-speed binary modulation provided by the DMDs. Furthermore, we substitute the four-step phase-shift algorithm with the three-step phase-shifting algorithm^34–38^ to reduce the number of the total measurements. In particular, we demonstrate a 20,000 Hz projection rate for FSI and capture 256 × 256-pixel dynamic scenes at a speed of 10 frames per second. The reported technique enables high-quality and high-speed imaging via single-pixel detectors. It may find broad imaging applications at wavebands that are not accessible for conventional 2-D image sensors.

## Method

**Figure 1:**
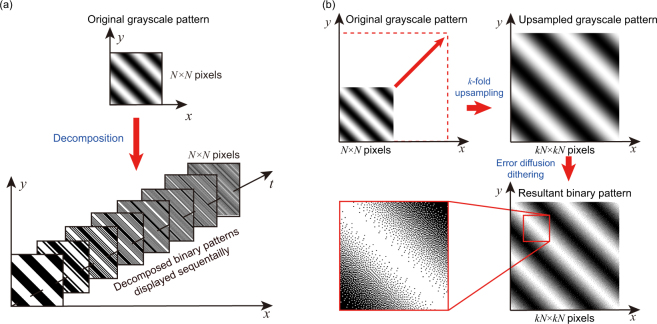
Fourier basis patterns generation by (**a**) temporal dithering and (**b**) spatial dithering.

The original FSI uses grayscale Fourier basis patterns for illumination. The intensity of each pattern is sinusoidal. Each Fourier basis pattern is characterized by its spatial frequency pair (*f*_*x*_, *f*_*y*_) and its initial phase *ϕ*:

1 $${P}_{\varphi }(x,y)=a+b\cdot \,\cos (2\pi {f}_{x}x+2\pi {f}_{y}y+\varphi ),$$

where (*x*, *y*) is 2-D Cartesian coordinates, *a* is the average intensity of the pattern, and *b* is the modulation depth. A typical FSI system uses a DMD for generating greyscale illumination patterns. DMDs have millions of mirrors and each mirror has only two states (‘ON’ and ‘OFF’). When a mirror is ‘ON’, it reflects the light towards the object to be illuminated; when the mirror is ‘OFF’, it reflects the light towards the other direction. Each mirror can be individually controlled and switch between the two states. Therefore, DMDs can generate various different binary (black-and-white) patterns, but can’t generate grayscale patterns directly. As shown in Fig. 1(a), to generate a grayscale pattern, DMD-based systems are designed to decompose each grayscale pattern into a sequence of binary patterns (also known as bitplanes). For example, a grayscale pattern with 256 gray levels (8-bit image) will be decomposed into 8 binary patterns (1-bit images). The resultant binary patterns are then displayed on the DMD sequentially in a predefined amount of time. As the time for displaying each binary pattern is very short, the quick switching of binary patterns visually results in a grayscale pattern. Each pixel’s intensity in the grayscale pattern is the temporally weighted (by the predefined amount of time) mean of the intensities of the 8 binary patterns at the same pixel. Such a scheme is termed temporal dithering. It can be seen that Fourier basis patterns generation via temporal dithering is at the expense of time (or temporal resolution). Even state-of-the-art DMDs can only generate 8-bit grayscale patterns at a rate of ~250 Hz which is far lower than the rate (~20,000 Hz) of binary patterns. Therefore, the original FSI is difficult to be applied for high-speed imaging even by using a high-speed DMD.

In order to achieve high-speed Fourier basis patterns generation using a DMD, we propose to employ image upsampling and Floyd-Steinberg error diffusion dithering method to generate binary Fourier basis patterns. With the generated binary patterns, we can make full use of the maximum illumination rate allowed by a DMD. To reconstruct an image with *N* × *N* pixels in Fig. 1(b), we first generate a complete set of grayscale Fourier basis patterns. The image resolution of each basis pattern is *N* × *N* pixels. We then apply upsampling to the grayscale patterns using the ‘bicubic’ image interpolation algorithm. The size of the upsampled patterns is *kN* × *kN* pixels. As such, each pixel in the original pattern will be represented by *k* × *k* pixels in the upsampled pattern. In other words, the upsampled pattern consists of *N* × *N* super pixels and each super pixel consists of *k* × *k* regular pixels, similar to *k* × *k* pixels binning^31^. Finally, we perform the error diffusion based dithering to the upsampled pattern and generate the binarized Fourier basis patterns.

Binarization causes quantization errors to every single pixel. The errors can be positive or negative. Error diffusion dithering spreads (adds) the residual quantization error of a pixel onto its neighboring pixels. If the quantization errors of a number of pixels are negative, the quantization error of the next pixel become more likely to be positive. As such, the average quantization error in a local area is close to zero. In our approach, we use higher-resolution illumination patterns (*kN* × *kN* pixels) to reconstruct a lower-resolution image (*N* × *N* pixels), the image reconstruction process implies a *k*-fold downsampling. As such, the quantization errors of every single pixel in the reconstructed image are averaged by *k* × *k* pixels. A larger *k* leads to smaller quantization errors and larger pixel size for each super pixel. However, larger super pixel results in lower achievable spatial resolution. It can be seen that Fourier basis patterns generation via the proposed spatial dithering is at the expense of spatial resolution. As spatial dithering is a nonlinear process, we have difficulties in giving a close-form expression. Instead, we will present numerical simulations in the following section to demonstrate that the combination of error diffusion dithering and image upsampling can eliminate the undesired quantization errors and achieve high-quality reconstructions.

In order to further speed up data acquisition process, we employ three-step phase-shifting illumination to reduce the number of measurements *M*. Three-step phase-shifting algorithm is widely used in fringe analysis^34–36^, optical sectioning^37^, and structured illumination microscopy^38^. Here we employ the three-step phase-shifting algorithm in Fourier coefficients acquisition. Fourier basis patterns with different spatial frequency pairs are generated according to Eq. (1). Each spatial frequency pair (*f*_*x*_, *f*_*y*_) corresponds to three different initial phases (*ϕ* = 0, 2*π*/3 and 4*π*/3 rad). As such, each complex-valued Fourier coefficient *Ĩ* can be obtained by illuminating three patterns and using the three corresponding responses (*D*_0_, *D*_2*π*/3_ and *D*_4*π*/3_) for calculation:

2 $$\tilde{I}({f}_{x},{f}_{y})=[2{D}_{0}({f}_{x},{f}_{y})-{D}_{2\pi /3}({f}_{x},{f}_{y})-{D}_{4\pi /3}({f}_{x},{f}_{y})]+\sqrt{3}{\rm{j}}\cdot [{D}_{2\pi /3}({f}_{x},{f}_{y})-{D}_{4\pi /3}({f}_{x},{f}_{y})].$$

The number of Fourier coefficients in Fourier domain is the same as the number of image pixels in spatial domain. Each Fourier coefficient is complex-valued. As the Fourier spectrum of a natural (real-valued) image is conjugate symmetric, we can obtain a pair of conjugate symmetric Fourier coefficients [*Ĩ*(*f*_*x*_, *f*_*y*_) and *Ĩ*(−*f*_*x*_, −*f*_*y*_)] with 3 measurements. Consequently, fully sampling an *N* × *N*-pixel object image consumes 1.5*N* ^2^ (=*N* × *N* × 3/2) measurements.

## Results

### Numerical simulations

**Figure 2:**
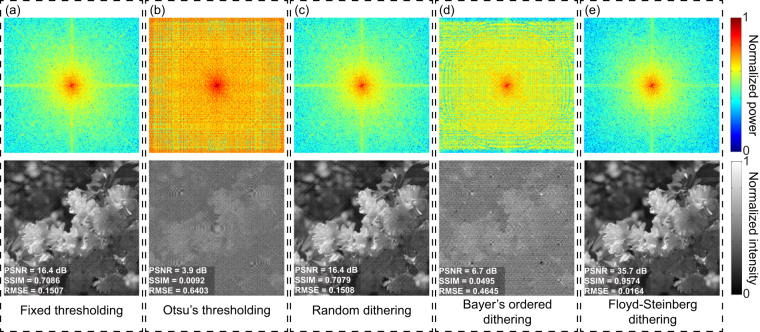
Reconstructed Fourier spectra and images for different binarization methods using the upsampling ratio *k* = 2: (**a**) fixed thresholding, (**b**) Otsu’s thresholding, (**c**) random dithering, (**d**) Bayer’s ordered dithering, and (**e**) Floyd-Steinberg error diffusion dithering.

In the first simulation, we explore different binarization methods that are used in Fourier basis patterns binarization. The binarization methods in comparison are (1) the fixed thresholding method, (2) Otsu’s thresholding, (3) random dithering, (4) Bayer’s ordered dithering, and (5) the Floyd-Steinberg error diffusion dithering. An image with 512 × 512 pixels is used as the original object image. In order to reconstruct a 256 × 256-pixel image, the Fourier basis patterns *P*(*x*, *y*) are generated according to Equation (1) where *a* = 0.5, *b* = 0.5, and spatial frequencies, *f*_*x*_, *f*_*y*_, range from 0, 1/256, 2/256, …, 255/256. The basis patterns are first upsampled with a ratio *k* = 2 using the ‘bicubic’ image interpolation algorithm and then binarized. The resultant responses of single-pixel detector, *D*_*ϕ*_, are simulated by

3 $${D}_{\varphi }=\sum _{x}\sum _{y}P(x,y)\cdot I(x,y),$$

where *P*(*x*, *y*) denotes a binarized Fourier basis pattern and *I*(*x*,*y*) is the object image. The Fourier spectrum of the image to be reconstructed is obtained according to Eq. (2). The final image is reconstructed by applying an inverse fast 2-D Fourier transform. To quantitatively evaluate the quality of reconstructions, we use peak signal-to-noise ratio (PSNR), structural similarity index (SSIM), and root mean square error (RMSE). Figure 2 shows the reconstructed Fourier spectra and images for different binarization methods. The result for the Steinberg-Floyd error diffusion dithering is clear without noticeable artifacts, much better than the results by the rest four methods in terms of reconstruction quality. The reconstructions by the other four binarization methods are drenched in noise and artifacts. The noise and artifacts are due to quantization errors caused by binarization. The results demonstrate that the spatial dithering is able to effectively eliminate quantization errors and produce high-quality reconstructions.

Without loss of generality, we also use 10 standard images from a public image database for testing^39^. We present the mean quality of the 10 reconstructed images for different binarization methods in Table 1.

**Table 1:** Quality of reconstructed images for different binarization methods.

Binarization method	PSNR	SSIM	RMSE
Fixed thresholding	15.0 dB	0.6576	0.1801
Otsu’s thresholding	2.2 dB	−0.0050	0.7866
Random dithering	15.0 dB	0.6565	0.1801
Bayer’s ordered dithering	5.0 dB	0.0382	0.5707
Floyd-Steinberg dithering	**34.1 dB**	**0.9285**	**0.0201**

**Figure 3:**
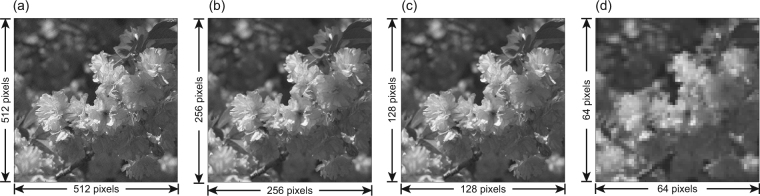
Simulation results with different upsampling ratios: (**a**) *k* = 1, (**b**) *k* = 2, (**c**) *k* = 4, and (**d**) *k* = 8.

In the second simulation, we test the influence of the upsampling ratio *k* on the quality of final reconstruction. The resolution of the basis patterns used in this simulation is (512/*k*) × (512/*k*) pixels so that the resolution of upsampled basis patterns is the same as that of the object image. The resolution of the reconstructed image is also (512/*k*) × (512/*k*) pixels, the same as that of the original basis pattern. The greyscale basis patterns are first upsampled using the ‘bicubic’ interpolation (with *k* = 1, 2, 4, and 8) and then binarized using Floyd-Steinberg error diffusion dithering. The simulation results are presented in Table 2 and Fig. 3.

**Table 2:** The quality of reconstructed images different upsampling ratios.

*k*	PSNR	SSIM	RMSE
1	21.5 dB	0.308	0.0839
2	34.4 dB	0.924	0.0191
4	38.8 dB	0.979	0.0115
8	42.6 dB	0.987	0.0074

The results demonstrate that larger sampling ratio *k* enables better performance in quantization errors elimination and therefore better reconstruction quality. As evidenced by Table 2 and Fig. 3, the quality of reconstruction improves remarkably when the value of *k* increases from 1 to 2, but improves slightly when *k* is larger than 2. On the other hand, the spatial resolution decreases as *k* increases. It turns out that there is a tradeoff between errors and resolution, and *k* = 2 is the ‘sweet point’ for this tradeoff.

### Experimental set-up

**Figure 4:**
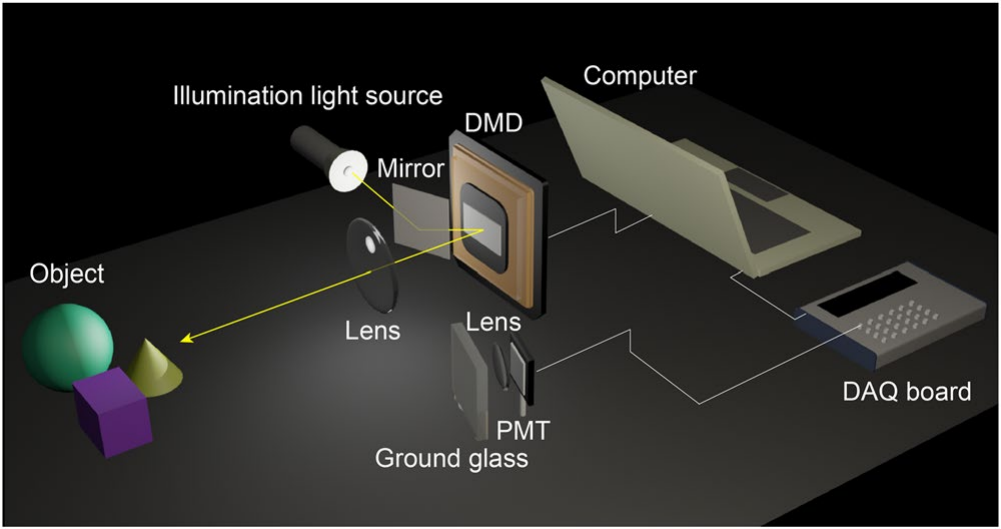
Experimental set-up.

The experimental set-up is shown in Fig. 4. The set-up consists of an illumination system, a detection system, and an object. The illumination system consists of a Texas Instruments DLP Discovery 4100 development kit, a lens system, and a 3-watt white LED. The DLP development kit is equipped with a 0.7-inch DMD which has 1024 × 768 micro mirrors. Each mirror is 13.6 × 13.6 μm^2^ in size. The LED continuously emits uniform light fields towards the mirror. With the mirror, light fields are reflected onto the DMD, then modulated by the DMD, and finally reflected onto the object. We make up a complex scene where a piece of A4 paper with a printed enlarged 1951 USAF resolution test pattern is used as the background. Through the lens system, clear illumination patterns are formed on the object surface. Note that the lens system is simply removed from a commercial digital projector (TOSHIBA T-90). The illumination light fields are scattered by the object and the resulting scattered light fields are collected by the detection system. The detection system consists of a photomultipliers tube (Thorlabs PMM01) which is used as a single-pixel detector, a collecting lens, a piece of ground glass, a custom amplification circuit, a data acquisition board [National Instruments USB-6343 (BNC)], and a computer. The maximum rate for analog input of the data acquisition board (DAQ) is 500,000 samples per second.

### Static imaging results

We employ three-step phase-shifting binary FSI to reconstruct a 256 × 256-pixel image of the object. The sampling ratio is 100%. As such, 256 × 256 complex-valued Fourier coefficients are to be sampled. We firstly generate a complete set of grayscale Fourier basis patterns. The number of patterns is 98,304 (=256 × 256 × 3/2). The division of 2 is for conjugate symmetry of the Fourier spectrum of real-value image. The image resolution of each pattern is 256 × 256 pixels. We then upsample the basis patterns by using ‘bicubic’ image interpolation algorithm with upsampling ratio *k* = 2 so that each upsampled pattern is represented by 512 × 512 pixels. Floyd-Steinberg error diffusion dithering is applied to all the patterns. For perfect reconstruction, the Fourier spectrum is fully sampled by projecting all patterns.

**Figure 5:**
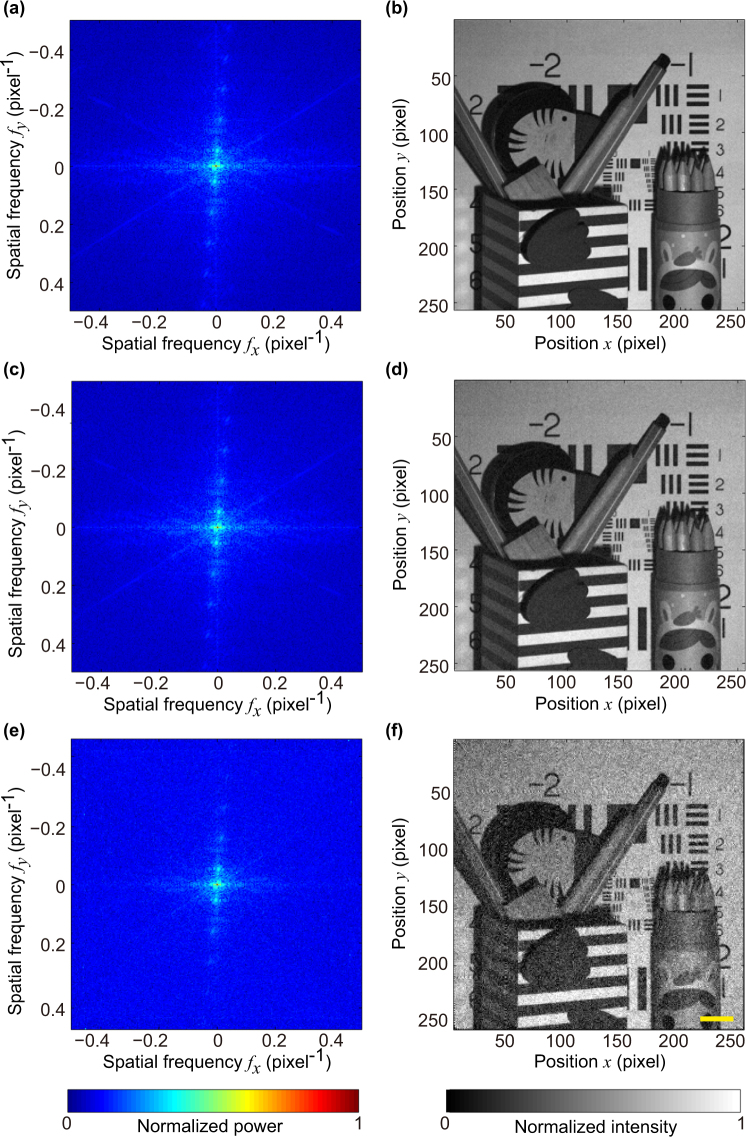
Static imaging for different illumination rates. The resolution of the reconstructed images is (**a**) Fourier spectrum sampled at illumination rate of 250 Hz and (**b**) corresponding reconstructed image; (**c**) and (**d**) at illumination rate of 2,000 Hz; (**e**) and (**f**) at illumination rate of 20,000 Hz. Note that we show the absolute value of the Fourier spectra on a logarithm scale to render them visible. No post processing has been applied to all reconstructed images. Scale bar = 2 cm.

Figure 5 shows the sampled Fourier spectra and the corresponding reconstructed images for different illumination rates: 250 Hz, 2,000 Hz, and 20,000 Hz. The illumination rate refers to the number of patterns projected onto the object per second by the DMD. The corresponding spatial information acquisition time is 394 seconds, 50 seconds, and 5 seconds, respectively. Figure 5(b) shows the reconstructed image for 250 Hz. The reconstruction is of high quality, for it presents fine details of the object and has not noticeable noise or artifacts. This high-quality reconstruction is due to the DMD operating at a relatively slow mode and resultant long acquisition time. As the utilized DAQ operates at the rate of 500,000 samples per second and the DMD operates at 250 Hz, the DAQ collects 2,000 samples for each illumination pattern. The number of samples is so large that random noise can be well evened out. Figure 5(d) shows the result for the illumination rate of 2,000 Hz. Although the illumination rate is 8-fold of the maximum rate for 8-bit grayscale patterns illumination, the reconstructed image is still visually as good as that acquired at 250 Hz. This result demonstrates that the proposed technique is capable of high-quality static imaging with an illumination rate higher than 250 Hz, the maximum rate for 8-bit grayscale patterns illumination allowed by the DMD. Figure 5(f) shows the reconstructed image for illumination rate of 20,000 Hz, the maximum allowable illumination rate for binary patterns. Although noticeable noise is presented in the reconstructed image, the quality is acceptable. When the DMD operates at 20, 000 Hz, there are only 25 samples that the DAQ can detect for each illumination pattern. In other words, the data acquisition rate of the utilized DAQ relatively low. We believe that the quality of reconstruction for 20,000 Hz might be improved by using a more advanced DAQ.

### Dynamic imaging results

**Figure 6:**
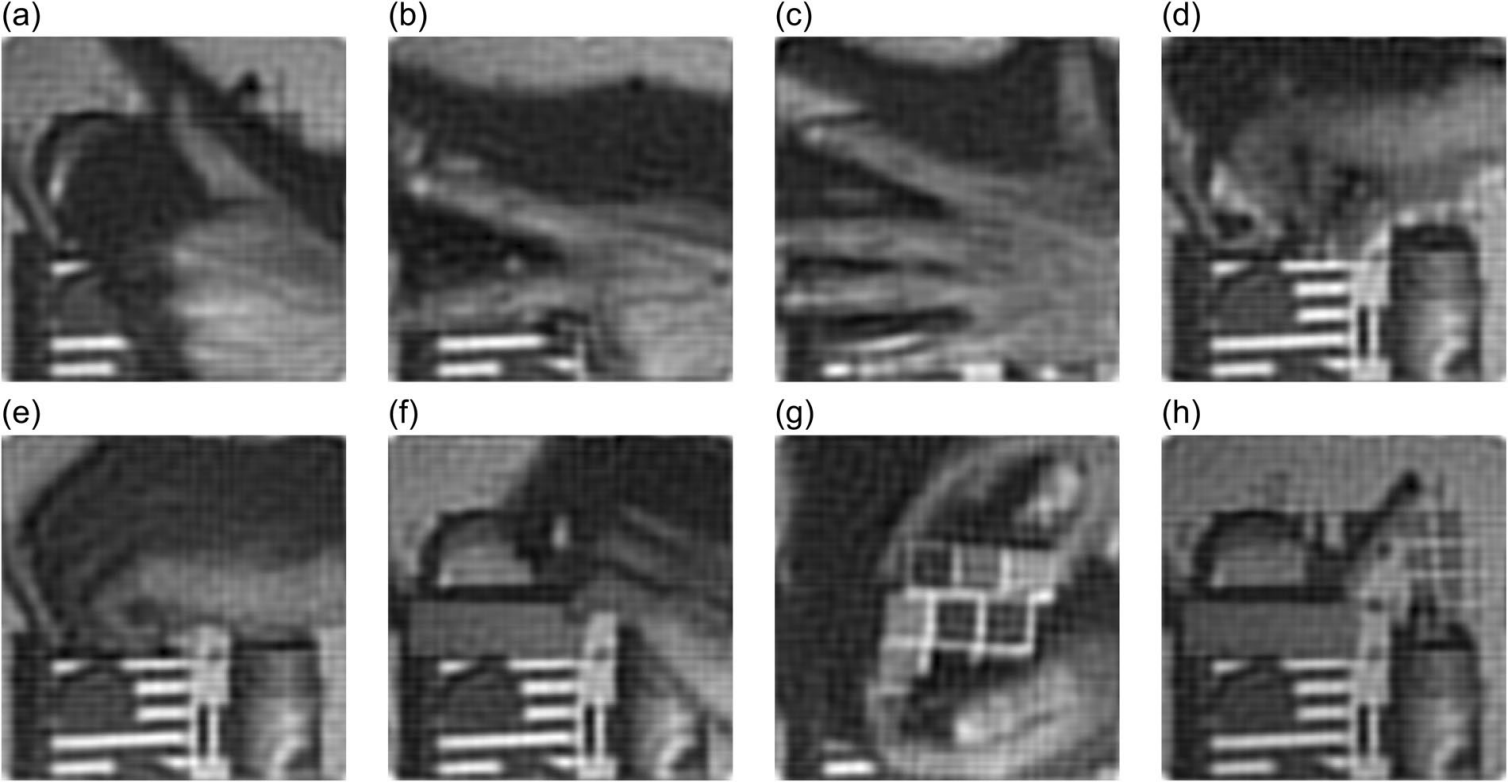
Dynamic imaging results. (**a**) to (**h**) show 8 of 167 images reconstructed. No post-processing has been applied to the reconstructed images. See Supplementary Video 1 for the complete video.

We finally apply the proposed technique for imaging dynamic scenes. In this experiment, the DMD operates at the maximum modulation rate of 20,000 Hz. Our aim is to capture a dynamic scene with 10 frames per second. The resolution is 256 × 256 pixels. To reduce the number of measurements, we utilize the prior that most spatial information of natural images is concentrated at the low spatial frequencies range in the Fourier domain. We perform sub-Nyquist sampling in image acquisition, acquiring only 1,332 coefficients (666 coefficient pairs, each of which takes 3 measurements) in the low-frequency range of the Fourier domain along a spiral path for each image (the detailed sampling strategy is described in refs^30,40^). Thus, each image takes 1,998 measurements, which allows us to capture ~10 images per second. The sampling ratio is ~2% (=1332/256^2^). We generate 1,998 binary Fourier basis patterns whose original resolution is 256 × 256 effective pixels, using *k* = 2 for upsampling and Floyd-Steinberg’s method for dithering. We capture 167 images and reconstruct a ~17-second video (see Supplementary Video 1). The video is created from the sequence of reconstructed images and the playback frame rate is set to be 10. No post-processing has been applied to the reconstructed images and the video. Although the ringing effect and motion blur are noticeable, some details in the reconstruction, the popping veins at the back of the human hand (see Fig. 6(a–c)) for example, are still well presented. The quality can be improved by utilizing the inter-frame redundancy or employing deconvolution as post processing.

## Discussion

We report a fast imaging technique that jointly employs image upsampling and an error diffusion dithering method to generate binary Fourier basis patterns for illumination. This strategy has two advantages. First, the illumination rate is two orders of magnitude higher than that of the original greyscale implementation (80-fold, 20,000 Hz achieved in our experiments, 250 Hz allowed by the DMD in 8-bit mode). Second, the binary patterns are free from gamma distortion (nonlinearity of gray levels). This strategy also has the two disadvantages. First, dithering causes quantization errors. Even the utilization of error diffusion dithering cannot perfectly eliminate the errors. We experimentally demonstrate that noises due to quantization errors are barely noticeable in the final reconstruction with the combination of the Floyd-Steinberg error diffusion dithering and 2-fold upsampling. Second, high-speed binary Fourier basis patterns generation is at the expense of spatial resolution. Defocusing technique might make the binary Fourier basis patterns more sinusoidal. However, defocusing also lead to the loss of high-frequency information of the illumination patterns.

We employ the three-step phase-shifting algorithm which brings an advantage of 25% fewer measurements than the four-step phase-shifting algorithm used in our previously proposed technique^17,30^. In principle, the two-step phase-shifting (*π*-shift) algorithm is able to acquire the complete Fourier spectrum of an image with as many measurements as the reconstructed pixels. In practice, however, the two-step phase-shifting algorithm is a direct method of measurement and therefore more sensitive to noise than the three-step phase-shifting and four-step phase-shifting algorithms.

The achievable highest acquisition rate depends on the rate of the utilized DMD, the response of the utilized detector and the sampling rate of the utilized DAQ. Higher acquisition rate might ease motion blur in dynamic imaging. However, higher acquisition rate leads to fewer photons detected and lower SNR, which would result in degeneration of quality. Thus, there is a tradeoff between speed and quality.

Adaptively sampling the most important (of largest modulus) Fourier coefficients would make the image acquisition process more efficient. The use of advanced sampling strategies^41,42^ in Fourier coefficients acquisition might allow further reduction of measurements. The proposed technique can also potentially be combined with the 3-D FSI technique^17^ to achieve real-time 3-D single-pixel imaging.

In conclusion, we successfully accelerate the FSI using binary patterns for illumination and develop a fast DMD-based single-pixel imaging system. We demonstrate a 20,000 Hz projection rate using the DMDs and capture 256 × 256-pixel dynamic scenes at a speed of 10 frames per second. The reported technique enables high-quality and high-speed imaging via single-pixel detectors. It may find broad imaging applications at wavebands that are not accessible using conventional 2-D image sensors.

## Electronic supplementary material

Supplementary Information

Supplementary Video S1

## References

[1] Webb RH (1996). Confocal optical microscopy. Rep. Prog. Phys..

[2] Huang D (1991). Optical coherence tomography. Science.

[3] Dimagno E (1980). Ultrasonic endoscope. Lancet.

[4] Oatley CW, Nixon WC, Pease RFW (1966). Scanning electron microscopy. Adv. Electron El. Phys..

[5] Pittman TB, Shih YH, Strekalov DV, Sergienko AV (1995). Optical imaging by means of two-photon quantum entanglement. Phys. Rev. A.

[6] Bennink RS, Bentley SJ, Boyd RW (2002). ‘Two-Photon’ coincidence imaging with a classical source. Phys. Rev. Lett..

[7] Shapiro JH (2008). Computational ghost imaging. Phys. Rev. A..

[8] Ferri F, Magatti D, Lugiato LA, Gatti A (2010). Differential ghost imaging. Phys. Rev. Lett..

[9] Sun B, Welsh SS, Edgar MP, Shapiro JH, Padgett MJ (2012). Normalized ghost imaging. Opt. Express..

[10] Welsh SS (2013). Fast full-color computational imaging with single-pixel detectors. Opt. Express..

[11] Chan WL (2008). A single-pixel terahertz imaging system based on compressed sensing. Appl. Phys. Lett..

[12] Watts CM (2014). Terahertz compressive imaging with metamaterial spatial light modulators. Nat. Photonics.

[13] Sun B (2013). 3-D Computational imaging with single-pixel detectors. Science.

[14] Howland GA, Lum DJ, Ware MR, Howell JC (2013). Photon counting compressive depth mapping. Opt. Express.

[15] Zhang Y (2016). 3D single-pixel video. J. Opt..

[16] Gong W (2016). Three-dimensional ghost imaging lidar via sparsity constraint. Sci. Rep..

[17] Zhang Z, Zhong J (2016). Three-dimensional single-pixel imaging with far fewer measurements than effective image pixels. Opt. Lett..

[18] Sun MJ (2016). Single-pixel three-dimensional imaging with time-based depth resolution. Nat. Commun.

[19] Edgar MP (2015). Simultaneous real-time visible and infrared video with single-pixel detectors. Sci. Rep..

[20] Bian L (2016). Multispectral imaging using a single bucket detector. Sci. Rep..

[21] Guo K, Jiang S, Zheng G (2016). Multilayer fluorescence imaging on a single-pixel detector. Biomed. Opt. Express.

[22] Rodríguez AD, Clemente P, Tajahuerce E, Lancis J (2016). Dual-mode optical microscope based on single-pixel imaging. Optics and Lasers in Engineering.

[23] Field JJ, Winters DG, Bartels RA (2016). Single-pixel fluorescent imaging with temporally labeled illumination patterns. Optica.

[24] Clemente P, Durán V, Tajahuerce E, Lancis J (2010). Optical encryption based on computational ghost imaging. Opt. Lett..

[25] Tajahuerce E (2014). Image transmission through dynamic scattering media by single-pixel photodetection. Opt. Express.

[26] Ma J (2009). Single-pixel remote sensing. IEEE Geoscience and Remote Sensing Letters.

[27] Ma J (2009). A single-pixel imaging system for remote sensing by two-step iterative curvelet thresholding. IEEE Geoscience and Remote Sensing Letters.

[28] Welsh SS, Edgar MP, Bowman R, Sun B, Padgett MJ (2015). Near video-rate linear Stokes imaging with single-pixel detectors. J. Opt..

[29] Duarte MF (2008). Single-pixel imaging via compressive sampling. IEEE Signal Process. Mag..

[30] Zhang Z, Ma X, Zhong J (2015). Single-pixel imaging by means of Fourier spectrum acquisition. Nat. Commun..

[31] Sun MJ, Edgar MP, Phillips DB, Gibson GM, Padgett MJ (2016). Improving the signal-to-noise ratio of single-pixel imaging using digital microscanning. Opt. Express.

[32] Zhang Z, Wang X, Zheng G, Zhong J (2017). Hadamard single-pixel imaging versus Fourier single-pixel imaging. Optics Express..

[33] Floyd R, Steinberg L (1976). An adaptive algorithm for spatial grey scale. Proc. Soc. Inf. Display.

[34] Wang Y, Zhang S (2012). Three-dimensional shape measurement with binary dithered patterns. Appl. Opt..

[35] Huang PS, Zhang S (2006). Fast three-step phase-shifting algorithm. Appl. Opt..

[36] Creath K (1988). Phase-measurement interferometry techniques. Progress in optics.

[37] Neil MA, Juškaitis R, Wilson T (1997). Method of obtaining optical sectioning by using structured light in a conventional microscope. Opt. Lett..

[38] Gustafsson, M. G. L. Surpassing the lateral resolution limit by a factor of two using structured illumination microscopy. *Journal of microscopy* **198**(2), 82–87 (2000). 10.1046/j.1365-2818.2000.00710.x 10810003

[39] http://www.imageprocessingplace.com/.

[40] Born, M. & Wolf, E. Principles of optics: electromagnetic theory of propagation, interference and diffraction of light. Pergamon Press, Cambridge (1959).

[41] Bian L (2014). Content adaptive illumination for Fourier ptychography. Opt. Lett..

[42] Bian L, Suo J, Hu X, Chen F, Dai Q (2016). Efficient single pixel imaging in Fourier space. J. Opt..

