# Supplementary material for: Fast Fourier single-pixel imaging via binary illumination
Source: Sci Rep. 2017 Sep 20;7:12029. doi: 10.1038/s41598-017-12228-3 (PMC5607233; doi:10.1038/s41598-017-12228-3)
Supplement: Supplementary file 1 — Supplementary Information [file 41598_2017_12228_MOESM1_ESM.pdf]

Fast Fourier single-pixel imaging via binary illumination: supplementary material

Zibang Zhang[1], Xueying Wang[1], Guoan Zheng[2], and Jingang Zhong[1,3,*]

[1]Department of Optoelectronic Engineering, Jinan University, Guangzhou, 510632, China
[2]Biomedical Engineering, University of Connecticut, Storrs, CT, 06269, USA
[3]Guangdong Provincial Key Laboratory of Optical Fiber Sensing and Communications, Jinan University, Guangzhou, 510632, China
[*]corresponding.tzjg@jnu.edu.cn

Supplementary Video S1

In this video we show the acquisition of a live scene using the proposed technique. The video is made up with a sequence of 167 reconstructed images. The frame rate is 10 and thus the duration of the video is ~17 seconds. The resolution of reconstructed images is 256×256 pixels. Each image is reconstructed from 1,998 measurements and therefore the sampling ratio is ~2% (1,332 of 256×256 complex-valued coefficients sampled in Fourier domain). No post processing has been applied to the reconstructed images.
